# CoCoView - A codon conservation viewer via sequence logos

**DOI:** 10.1016/j.mex.2022.101803

**Published:** 2022-07-29

**Authors:** Beatriz Rodrigues Estevam, Diego Mauricio Riaño-Pachón

**Affiliations:** Computational, Evolutionary and Systems Biology Laboratory (LabBCES), Center for Nuclear Energy in Agriculture, University of São Paulo, Piracicaba/SP, Brazil

**Keywords:** Consensus sequence, Information theory, Codons representation, Conserved patterns

## Abstract

Sequence logos are a simple way to display a set of aligned sequences, and they are useful to identify conserved patterns. Since their introduction, several tools have been developed for generating these representations at the single residue level (amino acids or nucleotides). We have developed a tool to build sequence logos of protein-coding sequences at the codon level, allowing more accurate analysis of coding-sequences as they represent synonymous and non-synonymous changes instead of showing only changes that imply on amino acid substitutions. We built CoCoView on top of the Logomaker Python API. It creates codon sequence logos from a multiple sequence alignment of protein-coding sequences. Some properties of the data and the generated logos can be controlled by the end-users, such as data redundancy, plot type and alphabet color.

• Split aligned sequences into codon positions;

• For each position compute codon frequency and information content;

• Use the computed information to plot the graphic.

## Graphical abstract

**Figure:**
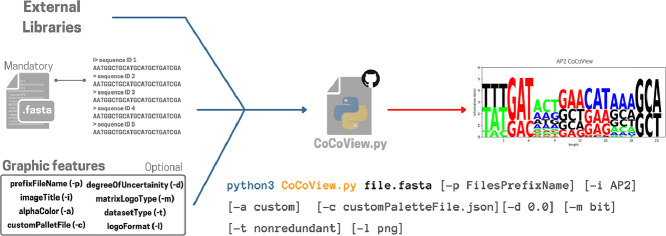

Specifications table

**Table:** 

Subject area	Bioinformatics

More specific subject area	Sequence analysis
Name of your method	CoCoView: A Codon Conservation Viewer via Sequence Logos
Name and reference of original method	Consensus sequence display via Sequence logos [1].
Resource availability	CoCoView.py and additional information are available on project's GitHub: https://github.com/labbces/CoCoView

## Method details

### Background information

Introduced by Schneider and Stephens (1990) sequence logos are composed of stacks of letters for each position of the multiple sequence alignment, following the conceptual bases of information theory [1], [2], [3]. The height of the stack (Rseq [Eq. 1]) is proportional to the conservation of the position; it is defined as the difference between the maximum possible entropy (Smax), defined as log2 of the number of symbols, and the observed entropy (H(l) [Eq. 2]). The height of a given base/amino acid/codon within the stack (Height [Eq. 3]), is measured by the product of its frequency and Rseq [Eq. 3] [1,4].

(1) $$R_{seq}\left(l\right)=S_{max}-H\left(l\right)=log_{2}N-H\left(l\right)$$

(2) $$H\left(l\right)=-\sum_{n=1}^{N}f\left(n,l\right)log_{2}f\left(n,l\right)$$

(3) $$Height\left(n,l\right)=f\left(n,l\right)R_{seq}\left(l\right)$$

Where f(n,l) represents the frequency of the symbol n (nucleotide, codon, or amino acid) at position l. N is the number of distinct symbols for a given alphabet (nucleotides, codons, or amino acids). Following this, Smax, for DNA and RNA that both have 4 nitrogenated bases, is log2(4) = 2 bits; for proteins with 20 different amino acids it is log2(20) ≈ 4.32 bits and for 64 codons it is log2(64) = 6 bits. Notice that when allowing for ambiguous nucleotides, the number of possible ‘codons’ would be higher, and so the Smax.

Codons have an important role in biology, they are the information unit in protein-coding sequences, during the process of translation. Changes in codon usage can have important functional consequences, for instance, even changes between synonymous codons can impact protein folding [5] or can affect the rate of protein elongation [6]. Analyzing codon usage on a positional basis allows the identification of consensus/conserved sequences and their variants in DNA regions that represent active, cleavage, and allosteric sites in proteins, and also to analyze regulatory regions, as in mRNA sites that enhance or repress protein translation [7] and mRNA splicing regions [8].

There is a lack of current and easy-to-use tools to visualize codon variation on a positional basis, as previous implementations are no longer available [9]. We developed CoCoView, exploiting Logomaker [10] to create codon sequence logos.

## Materials and methods

We developed CoCoView as a single python v3 script, tested on v3.7 and v3.9, to generate the codon sequence logos. It is available at https://github.com/labbces/CoCoView and runs on the command-line interface. CoCoView relies on some external libraries that should be installed in advance: argparse [11], pandas [12], matplotlib [13], logomaker [10], json [14], and biopython [15]. We are using Logomaker as a base due to its flexibility, and also because among other features, it offers the possibility to transform probability matrices into bit matrices and to define where each symbol or glyph will be located on the plot [10].

### Input

CoCoView only requires a file with aligned nucleotide sequences in FASTA format that must contain aligned sequences whose length is multiple of three, it assumes that the sequence starts with a complete codon. It also has some command-line switches that can alter the behavior of the program, we will describe these later. As output two files are produced, the matrix computed, either with bits or probabilities, which was used to build the logo and the sequence logo in either png or pdf format.

### Command-line arguments for CoCoView

Required, input FASTA file: “fastaFile”: CoCoView only requires a single input file. The script can only deal with single nucleotide symbols following the modern IUPAC nucleotide code nomenclature for incompletely specified bases [16]. Ambiguous nucleotides can pose problems to define the codons, so CoCoView allows the user to filter out sequences based on the fraction of ambiguous nucleotides present, using the argument “degreeOfUncertainty”, see below. We recommend using at least 40 sequences to avoid underestimation of entropy [4].

Optional, –prefixFileName: CoCoView produces two output files. One of them is a matrix that can have bits or probabilities (see –matrixLogoType) and that is used to build the codon logo. The other output file is the codon logo in figure format (see –logoFormat). The value of this argument is used as a prefix to create these two output files.

Optional, –imageTitle: This argument is a string that will appear as the title at the top of the sequence logo. If not provided by the user a title will be automatically generated from the input file name

Optional, –matrixLogoType: CoCoView builds the codon logo based on a matrix, which can be:

• a probability matrix: A matrix of N (rows) x M (columns), in which N are the codon positions in the multiple sequence alignment, and M are the different codons. Each cell has the proportion (probability) of a given codon in a given position. The sum of all codon proportions for a given position must add to 1.
• a bit matrix, default option: This is a transformation of the probability matrix, maintaining the same geometry, using the conceptual framework in equations 1 to 3. Each cell in the matrix represents the Height [Eq. 3] of a given codon in a given position, in bit units.

Optional, –alphaColor: CoCoView can use four different palettes of colors for the codon logos. Codons can be colored following the properties of their corresponding amino acids.The options are: “weblogo_protein (default)”, “charge”, “chemistry” and “hydrophobicity”.

Optional, –degreeOfUncertainty: Ambiguous nucleotides are allowed in the input sequence, however when they are present there is uncertainty about the amino acids they code for. With this argument the user can filter out sequences that have a proportion of ambiguous nucleotides greater than degreeOfUncertainty, using a floating-point number between 0 and 100. For example, a degreeOfUncertainty set to 30% will exclude all sequences of length equal to 12 that have at least 4 ambiguous nucleotides.

Optional, –datasetType: If duplicated sequences are present in the input dataset, setting this argument to ‘nonreduntant’ will remove duplicates from the analyses. This option is useful for small datasets. When very large datasets are used (thousands of sequences with hundreds/thousands of residues), users are advised to use third-party tools to generate non-redundant sequence sets, eg., cd-hit [17] or UCLUST [18]. Setting ‘nonreduntant’ may be of interest when the user wants to visualize less frequent codons. Default value ‘redundant’.

### Method validation - brief example

**Fig. 1:**
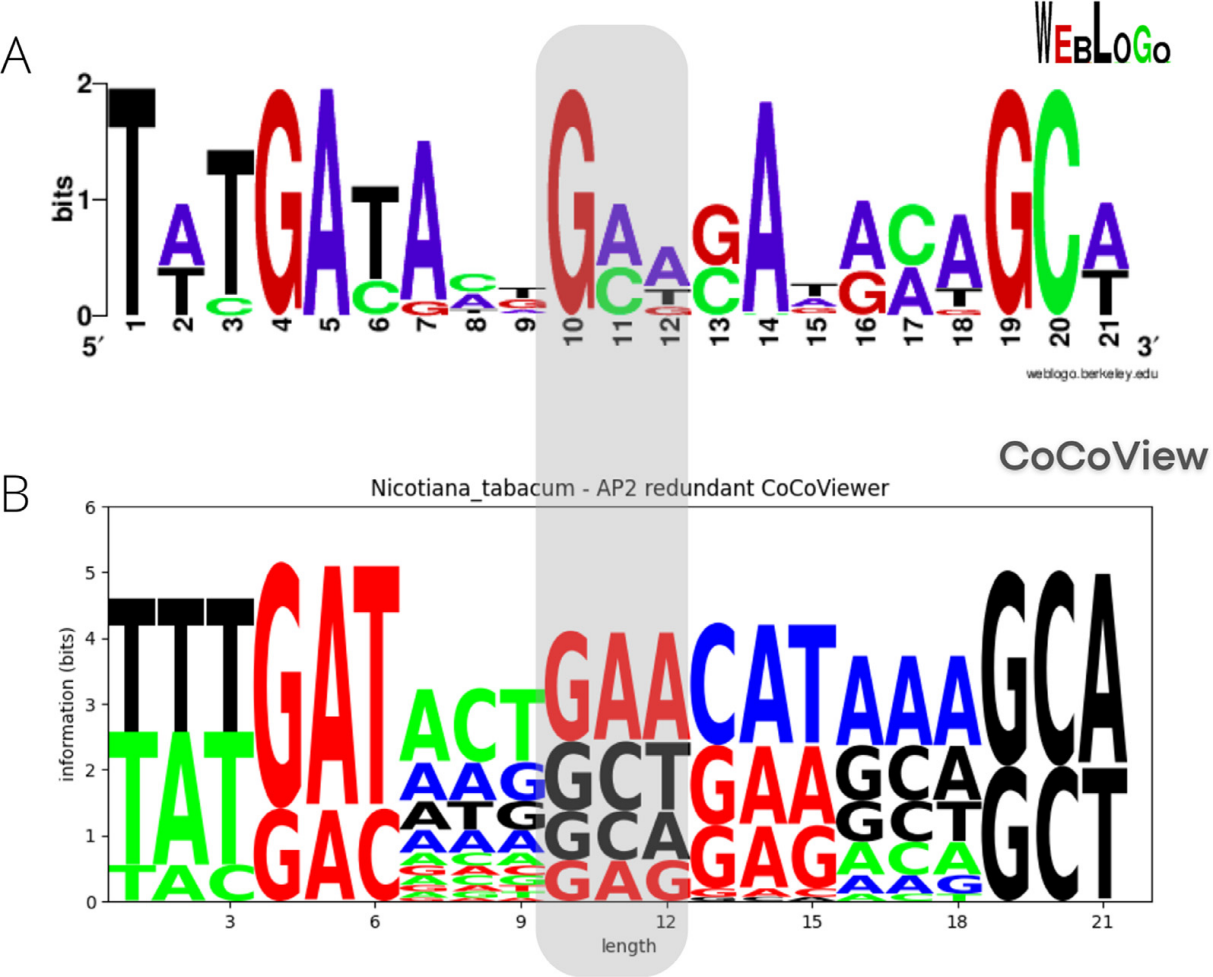
CoCoView logo based on a multiple sequence alignment of a region of AP2 transcription factor coding sequences from Nicotiana tabacum. (A) Sequence logo generated using WebLogo [4], representing a per-nucleotide analysis. (B) Sequence logo generated using CoCoView (per-codon analysis). A per-nucleotide analysis could erroneously suggest that some codons are common, which can be ruled out on a per-codon visualization. Exemplified by the codon “GAT”, at the position highlighted in gray on both sequence logos, which can be interpreted as a common codon in the per-nucleotide analysis. However, in the per-codon analysis, this codon does not occur at this position.

Transcription factors are proteins that bind DNA and regulate the expression of target genes. AP2 is a transcription factor involved in the regulation of growth and development, fruit ripening, defense response, and metabolism in plants [19]. In order to illustrate the benefits of a per-codon variation representation, we generated sequence logos using WebLogo [4] (per nucleotide analysis, Fig. 1A) and CoCoView (per codon analysis) for a region of the multiple sequence alignment of the coding sequences of AP2 from Nicotiana tabacum (Fig. 1B). In Fig. 1A, please note positions 10th to 12th, which represent the 4th codon of that region of the CDS, one could incorrectly draw the conclusion that the triplet “GAT '' is common at that position, based on the conservation of the individual nucleotides. However, when looking at the sequence logo based on condons on Fig. 1B, it is clear that “GAT'' is not common at all at this position.

## Conclusion

Here we presented CoCoView, a method to construct sequence logos using codons, which allows for a more detailed analysis of sequence conservation.

## CRediT authorship contribution statement

**Beatriz Rodrigues Estevam:** Software, Writing – original draft, Writing – review & editing. **Diego Mauricio Riaño-Pachón:** Conceptualization, Software, Resources, Writing – review & editing, Supervision, Project administration, Funding acquisition.

## Declaration of Competing Interest

The authors declare that they have no known competing financial interests or personal relationships that could have appeared to influence the work reported in this paper.
